# Berberine hydrochloride combination therapy and efflux pump regulation mechanism ameliorates antimicrobial resistance of *Acinetobacter baumannii*

**DOI:** 10.1128/spectrum.02565-25

**Published:** 2026-03-10

**Authors:** Jia Wang, Ye Kuang, Hongchao Zou, Chuanmei Peng, Yuanyuan Zhou, Xiang Zhang, Zidan Hu, Sulian Chen, Lei Feng

**Affiliations:** 1 Department of Medical Laboratory, Yan'an Hospital Affiliated To Kunming Medical University 619366, Kunming, China; 2 Department of Dermatology, Yan'an Hospital Affiliated To Kunming Medical University 619366, Kunming, China; Tel Aviv Sourasky Medical Center, Tel Aviv, Israel

**Keywords:** berberine hydrochloride, *Acinetobacter baumannii*, drug resistance, efflux pump

## Abstract

*Acinetobacter baumannii*, a major pathogen that causes nosocomial infections, exhibits drug resistance. Efflux pumps are critical in developing multidrug resistance. Berberine hydrochloride exhibited inhibitory effects against bacteria and toxins, demonstrating antibacterial efficacy. However, there is limited research on berberine hydrochloride combined with multiple antibiotics through regulation of efflux pumps in multidrug-resistant *A. baumannii*. We evaluated the antibacterial effect of berberine hydrochloride in combination with multiple antibiotics, explored the resistance mechanisms regulated by efflux pumps, and analyzed the differential expression of active efflux pump genes (*adeR*, *adeS*, *adeJ*, *adeM*, *adeG*, and *adeB*) before and after berberine hydrochloride exposure, to provide new therapeutic strategies for managing multidrug-resistant *A. baumannii* infections. One hundred non-repetitive *A. baumannii* strains were isolated from patients with respiratory infections. The microdilution method was used for the determination of minimal inhibitory concentration (MIC) and the checkerboard method for the determination of the fractional inhibitory concentration (FIC) index. Reverse transcription polymerase chain reaction quantified efflux pump gene expression in sensitive and resistant strains before and after berberine hydrochloride treatment. Strains showing significant differences were selected for transcriptomic sequencing. Berberine hydrochloride showed synergistic effects with nine antibiotics, the strongest being amikacin, meropenem, and cefepime. Resistance reversal occurred especially with levofloxacin. *adeJ and adeB* expression differed significantly between sensitive and resistant strains. Berberine hydrochloride induced notable changes in *adeS*, *adeG*, *adeB*, and *adeR*. Transcriptome sequencing revealed the significant expression of *adeA*, *adeN*, and *adeT2*. Berberine hydrochloride enhances antibiotic sensitivity and reverses resistance by regulating efflux pump gene expression, providing a theoretical foundation for improved treatment.

**IMPORTANCE:**

This study investigates multidrug-resistant *Acinetobacter baumannii* (MDRAB) strains isolated from clinical infections using the microplate dilution method to evaluate the antibacterial efficacy of berberine in combination with various antibiotics. The research simultaneously analyzes the differential expression levels of active efflux pump genes before and after berberine treatment. The findings aim to identify novel therapeutic strategies for MDRAB, explore the drug resistance mechanisms mediated by efflux pumps in AB, and elucidate berberine’s role in reversing AB resistance through efflux pump regulation. These insights provide theoretical foundations for improving cure rates in MDRAB-infected patients and advancing drug target design.

## INTRODUCTION

*Acinetobacter baumannii* is a clinically prevalent nosocomial bacterium and a major pathogen causing pneumonia, sepsis, meningitis, urinary tract infections, and wound infections. It is often associated with a high mortality rate. In recent years, diverse resistance mechanisms of *A. baumannii* have attracted extensive attention as the prevalence of *A. baumannii* infections has increased (1). The resistance mechanisms of *A. baumannii* can be grouped into three main categories: regulation of antibiotic transmembrane transport (decreasing porin permeability or enhancing efflux), modification of antibiotic targets, and enzymatic inactivation of antibiotics (2). Among these, the regulation of efflux pump mechanisms is critical for intracellular drug retention and therapeutic strategies. Evidence shows that the AdeABC efflux pump in *A. baumannii* promotes resistance by enhancing the efflux of aminoglycosides, certain β-lactams, fluoroquinolones, tetracyclines, tigecyclines, macrolides, chloramphenicol, and trimethoprim. Therefore, antibiotic resistance and cell growth in *A. baumannii* are closely associated with efflux pumps. Inappropriate and extensive use of antibiotics has facilitated the dissemination of multidrug-resistant (MDR) *A. baumannii* (MDRAB) within microbial communities, leading to a notable increase in MDRAB infections (3). The World Health Organization has recently categorized carbapenem-resistant *A. baumannii* as a critical priority pathogen in urgent need of new antibiotics (4); therefore, there is an urgent need to discover new antibacterial agents against MDRAB and explore broad antimicrobial therapies and/or combination antimicrobial therapies for the management of MDRAB infections.

Berberine hydrochloride (BBH), an isoquinoline alkaloid extracted from *Coptidis rhizoma* and other *Berberis* species, has been used in China for >2,000 years as an antibacterial agent for gastroenteritis, abdominal pain, and diarrhea. With advances in research, the antibacterial mechanism of BBH has progressively been elucidated (5). The complex composition of traditional Chinese medicine (TCM) makes bacteria less prone to developing resistance, positioning BBH as a research hotspot for alternative therapies against MDRAB (6). Studies have shown that berberine and its derivatives are effective antibacterial agents against methicillin-resistant *Staphylococcus aureus* and *Pseudomonas aeruginosa* (7). However, there is a paucity of research on the efficacy of BBH combined with multiple antibiotics against MDRAB and its mechanism for ameliorating resistance through the regulation of *A. baumannii* efflux pumps.

We aimed to evaluate the *in vitro* synergistic effect of BBH combined with multiple commonly used antibiotics against MDRAB and to explore the mechanism by which BBH reverses *A. baumannii* resistance through efflux pump regulation, thereby providing a scientific basis for TCM-based therapy against MDRAB infections and offering novel insights for clinical management to address the current therapeutic limitations of MDRAB.

## MATERIALS AND METHODS

### Strain source

A total of 50 *A. baumannii* strains were isolated from bronchoalveolar lavage fluid and endotracheal aspiration specimens obtained from patients clinically diagnosed with pulmonary infections at Yan'an Hospital, affiliated with Kunming Medical University, between December 2022 and December 2023. All clinical strain isolates used in this study were collected from a single tertiary care institution, and all strains were identified as *A. baumannii* using matrix-assisted laser desorption/ionization time-of-flight mass spectrometry (MALDI-TOF MS) and a compact automated microbial identification and susceptibility testing system (VITEK 2). The isolates were subsequently categorized into 50 drug-sensitive and 50 drug-resistant strains. Antimicrobial susceptibility results were interpreted according to the Clinical and Laboratory Standards Institute (CLSI) guidelines applicable to that year. Three quality control strains, *Escherichia coli* (ATCC 25922), *Pseudomonas aeruginosa* (ATCC 27853), and *Staphylococcus aureus* (ATCC 25923), were obtained from the National Center for Clinical Laboratories, Ministry of Health. MDRAB was defined according to established criteria, and all strains were stored at −70°C for future use. This study was approved by the Yan'an Hospital Affiliated to Kunming Medical University ethics committee (Approval No.: 2021-038-01; Date: 25 June 2021).

### Main equipment and consumables

The following instruments and reagents were used in this study: a mass spectrometer (*Bruker MALDI Biotyper IVD*, Bruker Corporation, Billerica, MA, USA); a VITEK 2 Compact instrument (*VITEK 2 Compact 60*, bioMérieux, Marcy-l'Étoile, France); antimicrobial susceptibility testing reagents (Kangtai Biotechnology Co., Ltd., Wenzhou, China); custom-designed primers (synthesized by Beijing Bomaide Gene Technology Co., Ltd., Beijing, China); cation-adjusted Mueller–Hinton broth (Haibo Biotechnology Co., Ltd., Qingdao, China); an incubator (Model BJPX-400, Shandong BioBase Biological Industry Co., Ltd., Jinan, China); a vortex mixer (*Mini Vortex Mixer H-1*, Shanghai Jingke Vortex Mixer, Guangzhou, China); an autoclave (GR85DP, Zhiwei Instruments Co., Ltd., Xiamen, China); a centrifuge (DT4, Xiangyi Centrifuge, Hunan Xiangyi, Hunan, China); and a nucleic acid amplification instrument (Stream SP96, Daan Gene Co., Ltd., Sun Yat-sen University, Guangzhou, China).

### BBH combination antibiotic assays

#### MIC determination of BBH

The minimal inhibitory concentration (MIC) of BBH was determined *in vitro* using a microdilution method. The culture medium, conditions, and procedures were performed according to the CLSI-M100 (current edition) and CLSI-M07-A9 guidelines. Berberine was dissolved in 1% dimethyl sulfoxide to prepare a 68.2 mg/mL stock solution, diluted in deionized water, and filtered through a 0.22 µm membrane. 1% DMSO has almost no antibacterial effect. The berberine concentrations tested ranged from 8 to 1,024 µg/mL. The plates were incubated at 37°C for 24 h, and the MIC was defined as the lowest concentration that inhibited visible bacterial growth. When the MIC was outside the test range or undetectable in the combination assays, the concentration gradients were adjusted until measurable MIC values were obtained (6).

#### BBH combination susceptibility testing

Checkerboard assays were performed to evaluate the combined activity of nine antibiotics (amikacin, tetracycline, ceftazidime, ciprofloxacin, cefepime, imipenem, meropenem, levofloxacin, and tigecycline) and BBH. The checkerboard dilution method involves first determining the MICs of the individual antibiotics, such as drug A and drug B, against the target bacterial strain. Subsequently, a series of dilutions for the combination assay is established based on these MIC values. Typically, seven twofold serial dilutions are selected, with the highest concentration of each antibiotic set at twice its respective MIC. This approach ensures a systematic evaluation of potential synergistic, additive, or antagonistic interactions between the drugs. The bacterial suspension reached 7.5 × 10^5^ CFU/mL per well. The plates were incubated at 35°C for 20–24 h, and the MIC was determined as the lowest concentration without visible growth.

The interaction between antibiotics and BBH was assessed using the fractional inhibitory concentration (FIC) index (7). The calculation formula for the FIC index is MIC (A in combination)/MIC (A alone) + MIC (B in combination)/MIC (B alone). An FIC of ≤0.5 indicates a synergistic effect, meaning that the antibacterial activity of the combination was significantly greater than that of either drug alone. An FIC between >0.5 and ≤1 denotes an additive effect, suggesting a slight increase in antibacterial activity compared to the individual drugs. An FIC between >1 and ≤2 indicates an indifferent effect, in which the activity of each drug remains unaffected by the other. Finally, an FIC greater than 2 signifies an antagonistic effect, implying that one drug reduces the activity of the other (8).

#### Exposure passage assay of BBH

Based on the FIC values obtained from the berberine combination drug sensitivity test, 15 MDR bacterial strains demonstrating relatively strong synergistic effects were selected. These strains were cultured in liquid medium containing berberine at 1/2 the MIC for five successive passages to establish the exposure group. The corresponding preserved MDR strains that were not exposed to berberine-containing medium served as the non-exposure group. Each experiment was conducted in triplicate.

### RNA isolation and efflux pump gene expression analysis

RNA was extracted using an Omega bacterial RNA kit according to the manufacturer’s instructions. ATCC 19699 served as the reference strain, with *16S rRNA* as the reference gene. The expression of *adeR*, *adeS*, *adeJ*, *adeM*, *adeG*, and *adeB* was quantified in sensitive and resistant strains and in the exposure and non-exposure groups using reverse transcription PCR. The primer sequences used are listed in Table 1. Triplicate reactions were performed for each gene, and the mean Ct values were calculated. The relative expression level of the target gene was calculated using the formula log_2_ (2^−ΔCt^), where ΔCt = Ct value of the target gene − Ct value of the reference gene 16S rRNA, which was stably expressed.

**TABLE 1:** Primer sequences^*a*^

Primer name	Primer	Sequence (5′ to 3′)	Product length (bp)
*adeB*	F	TTAACGATAGCGTTGTAACC	541
	R	TGAGCAGACAATGGAATAGT	
*adeR*	F	ACTACGATATTGGCGACATT	447
	R	GCGTCAGATTAAGCAAGATT	
*adeS*	F	TTGGTTAGCCACTGTTATCT	544
	R	AGTGGACGTTAGGTCAAGTT	
*adeJ*	F	ATTGCACCACCAACCGTAAC	453
	R	TAGCTGGATCAAGCCAGATA	
*adeM*	F	GTAGGTGTAGGCTTATGGA	303
	R	GTACCGAAGTGACTGAAAT	
*adeG*	F	TTCATCTAGCCAAGCAGAAG	468
	R	GTGTAGTGCCACTGGTTACT	
*16S rRNA*	F	GGAGGAAGGTGGGGATGACG	241
	R	ATGGTGTGACGGGCGGTGTG	

^*a*^ AdeB/J/M/G, AdeB/J/M/G efflux pump protein; adeR, AdeR regulatory protein; adeS, AdeS sensory kinase protein.

### Transcriptome sequencing

Transcriptome analysis was performed by Qingke Biotechnology (Beijing, China). Total RNA was extracted from the samples using the cetyltrimethylammonium bromide method, and contaminating genomic DNA was subsequently removed. High-quality RNA was used for library preparation. rRNA was depleted using the RiboCop rRNA Depletion Kit for Mixed Bacterial Samples (Lexogen, USA). The remaining mRNA was randomly fragmented into ~200 bp fragments, and double-stranded cDNA was synthesized using random primers. RNA libraries were constructed using the Illumina Stranded mRNA Prep Ligation kit. Paired-end RNA-seq was performed on the Illumina NovaSeq 6000 platform (or a comparable model). Bioinformatic analysis was conducted using the sequencing data generated, and DEGs were identified using DESeq2.

### Molecular docking

Molecular docking was employed to evaluate interactions between berberine chloride and efflux pump-associated proteins AdeB, AdeJ, AdeR, and AdeS. Protein structures were sourced from the PDB database or generated via homology modeling. All targets and ligands were processed using AutoDockTools (v 1.5.7), including charge assignment and format conversion. Docking simulations were performed using AutoDock (v 1.5.7) software. The resulting binding poses were visualized in PyMOL (v 4.60), and key intermolecular interactions were analyzed using Discovery Studio 2019.

### Statistical analysis

Statistical analyses were performed using IBM SPSS Statistics version 27 (IBM Corp., Armonk, NY, USA). The Wilcoxon test was used for non-normally distributed data, and the Student’s *t*-test was used for normally distributed data. Each strain was provided with two replicate wells, and each group of experiments was repeated three times. Statistical significance was set at *P* < 0.05.

## RESULTS

### *In vitro* synergistic effects of BBH combined with nine antibiotics

BBH demonstrated varying degrees of synergistic effects in combination with the nine antibiotics. The synergism rates exceeded 40.0% for amikacin, levofloxacin, meropenem, and cefepime, with the most pronounced synergistic effects observed for amikacin, meropenem, and cefepime (46.0%). The synergistic activities of tetracycline and ciprofloxacin were <30.0% (28.0% and 24.0%, respectively). The combined synergistic and additive effects of levofloxacin, ceftazidime, cefepime, and imipenem exceeded 98%, with levofloxacin and cefepime exhibiting 100.0% synergistic and additive effects. Berberine showed a 28.0% indifference to tigecycline, and no antagonistic effects were identified for any drug combination (Table 2). Furthermore, when berberine was used in combination with antibiotics, resistance reversal was observed in certain strains against levofloxacin, tetracycline, ciprofloxacin, ceftazidime, and meropenem, with the most significant resistance reversal effect observed for levofloxacin (Table 3).

**TABLE 2:** Partial fractional inhibitory concentration of berberine and nine antibiotics^*a*^

Name of drug	FIC ≤ 0.5	0.5 < FIC ≤ 1	1 < FIC ≤ 2	2 ≤ FIC	Total	Synergy (%)	Additivity (%)	Irrelevance (%)	Antagonism (%)	Synergy and additivity (%)	Irrelevance and antagonism (%)
BBH+AMK	23	23	4	0	50	46.00	46.00	8.00	0.00	92.00	8.00
BBH+CAZ	16	33	1	0	50	32.00	66.00	2.00	0.00	98.00	2.00
BBH+CIP	11	34	5	0	50	22.00	68.00	10.00	0.00	90.00	10.00
BBH+FEP	23	27	0	0	50	46.00	54.00	0.00	0.00	100.00	0.00
BBH+IPM	15	34	1	0	50	30.00	68.00	2.00	0.00	98.00	2.00
BBH+LVX	22	28	0	0	50	44.00	56.00	0.00	0.00	100.00	0.00
BBH+MEM	22	24	4	0	50	44.00	48.00	8.00	0.00	92.00	8.00
BBH+TGC	14	21	15	0	50	28.00	42.00	30.00	0.00	70.00	30.00
BBH+TET	14	29	7	0	50	28.00	58.00	14.00	0.00	86.00	14.00

^*a*^ FIC, fractional inhibitory concentration; BBH, berberine hydrochloride; AMK, amikacin; CAZ, ceftazidime; CIP, ciprofloxacin; FEP, cefepime; IPM, imipenem; LVX, levofloxacin; MEM, meropenem; TGC, tigecycline; TET, tetracycline.

**TABLE 3:** Reversal of antibacterial drug resistance of some strains by BBH^*a*^

Antibacterials	MIC of antibacterials (μg/mL)		MIC of berberine (μg/mL)	
	Monotherapy	Combination with BBH	Monotherapy	Combination with BBH
TET	16	2–4	512	32
MEM	8	2	256	32
LVX	8	1–2	256	64
CIP	4	1	256	64
CAZ	64	4	256	64
AMK	64	16	256	32

^*a*^ BBH, berberine hydrochloride; AMK, amikacin; CAZ, ceftazidime; CIP, ciprofloxacin; LVX, levofloxacin; MEM, meropenem; TET, tetracycline; MIC, minimum inhibitory concentration; combination, the group consists of six different antibiotics each combined with BBH.

### Efflux pump expression level differences

#### Efflux pump expression levels in the sensitive and resistant groups

Relative mRNA expression levels of efflux pump genes were detected in the two groups, and statistical analysis showed significant differences in the expression of adeJ and adeB between the sensitive and resistant groups of A. baumannii (*P* < 0.05, statistically significant) (Table 4).

**TABLE 4:** Expression differences of efflux pumps in the sensitive and resistant groups^*a*^

Gene	*adeB*	*adeR*	*adeS*	*adeJ*	*adeM*	*adeG*
*P* value	0.001	0.071	0.049	0.001	0.081	0.724

^*a*^ AdeB/J/M/G, AdeB/J/M/G efflux pump protein; adeR, AdeR regulatory protein; adeS, AdeS sensory kinase protein.

#### Molecular docking simulation of berberine with efflux pump-related proteins AdeB, AdeJ, AdeR, and AdeS

**Fig 1:**
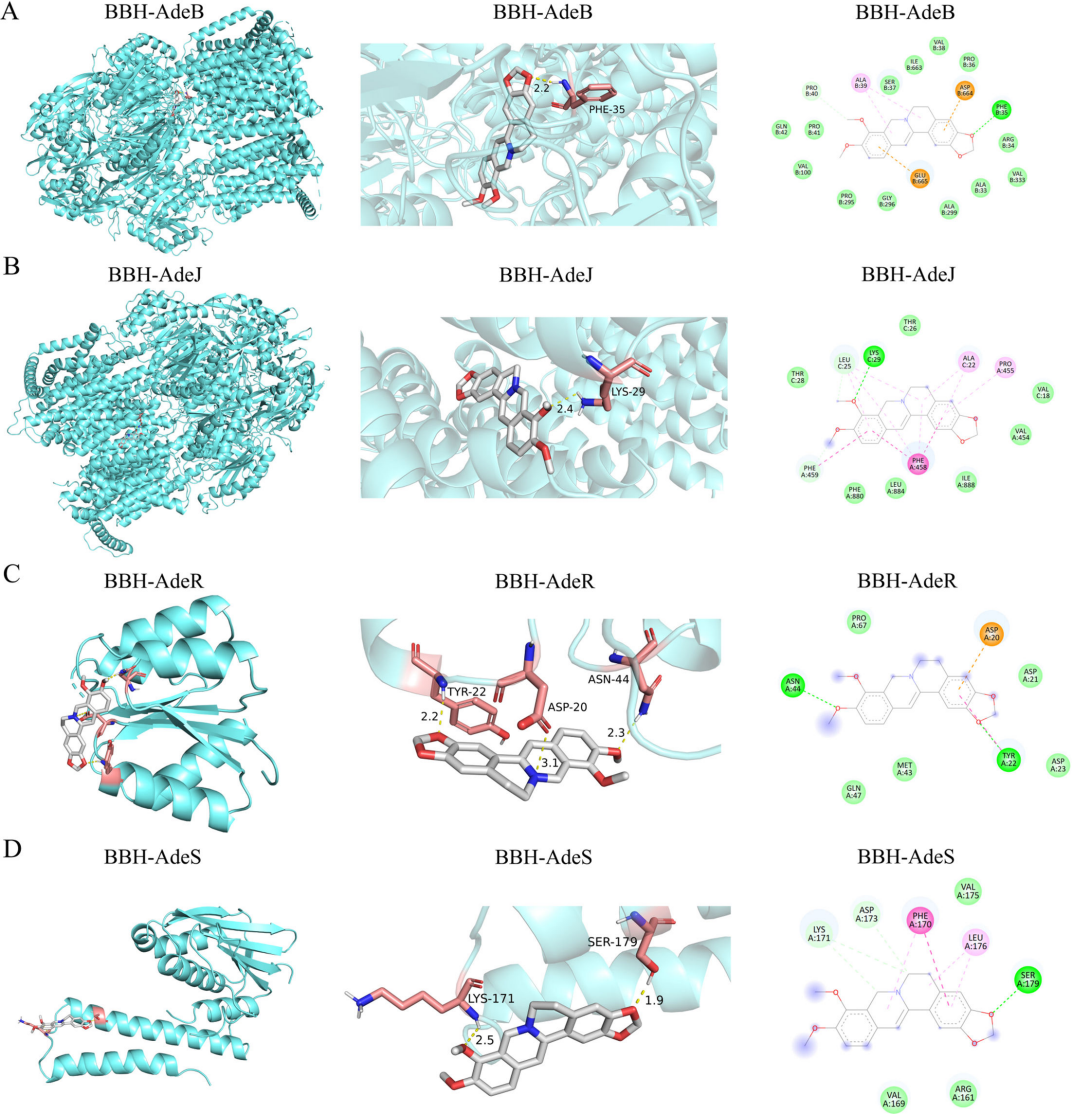
Molecular docking simulation of BBH with efflux pump-related proteins AdeB, AdeJ, AdeR, and AdeS. (**A**) BBH-AdeB. Left panel: the overall docking situation at the macro level was shown. The blue part represents the large protein molecule, while the white-blue ring-shaped structure indicates the small ligand compound molecule. Middle panel: an enlarged display of the docking details. The pink part represents the amino acid residues of the receptor protein, the yellow lines represent the hydrogen bonds that are bound, and the numbers represent the lengths of the hydrogen bonds. Right panel: the 2D visualization of the docking results, where the colors of the circles represent different interaction types, and the labels inside the circles are the residue numbers of the proteins. Residues with lines indicate that they have formed a definite interaction with the ligand. (**B**) BBH-AdeJ. (**C**) BBH-AdeR. (**D**) BBH-AdeS. adeB, AdeB efflux pump protein; adeG, AdeG efflux pump protein; adeR, AdeR regulatory protein; adeS, AdeS sensory kinase protein.

Based on the significant differential expression of efflux pump-associated genes identified in both the susceptibility comparison and the berberine exposure experiments, molecular docking was performed to further clarify the potential interaction between berberine chloride and key components of the Ade efflux systems. Berberine chloride showed stable binding to all four tested proteins (AdeB, AdeJ, AdeR, and AdeS), with the strongest affinities observed for the pump transporters AdeJ and AdeB, displaying binding energies of –6.71 and –6.02 kcal/mol, respectively. Moderate binding was also detected for the regulatory proteins AdeR and AdeS (–4.60 and –4.89 kcal/mol). These results agree with our RT-PCR data, in which adeB and adeJ exhibited significant differences between susceptible and resistant isolates, and adeB, adeR, adeS, and adeG were markedly altered following berberine exposure. Together with the transcriptomic findings showing differential expression of multiple RND-type efflux pump genes (including adeA, adeN, and adeT2), the docking results support the notion that berberine chloride may directly target both efflux pump transporters and their regulatory elements. This molecular interaction provides a plausible mechanistic explanation for the enhanced antibiotic susceptibility and reversal of resistance—particularly toward levofloxacin, meropenem, and amikacin—observed in our combination assays (Fig. 1A through D).

#### Efflux pump gene expression levels in the drug-resistant group before and after BBH exposure

**Fig 2:**
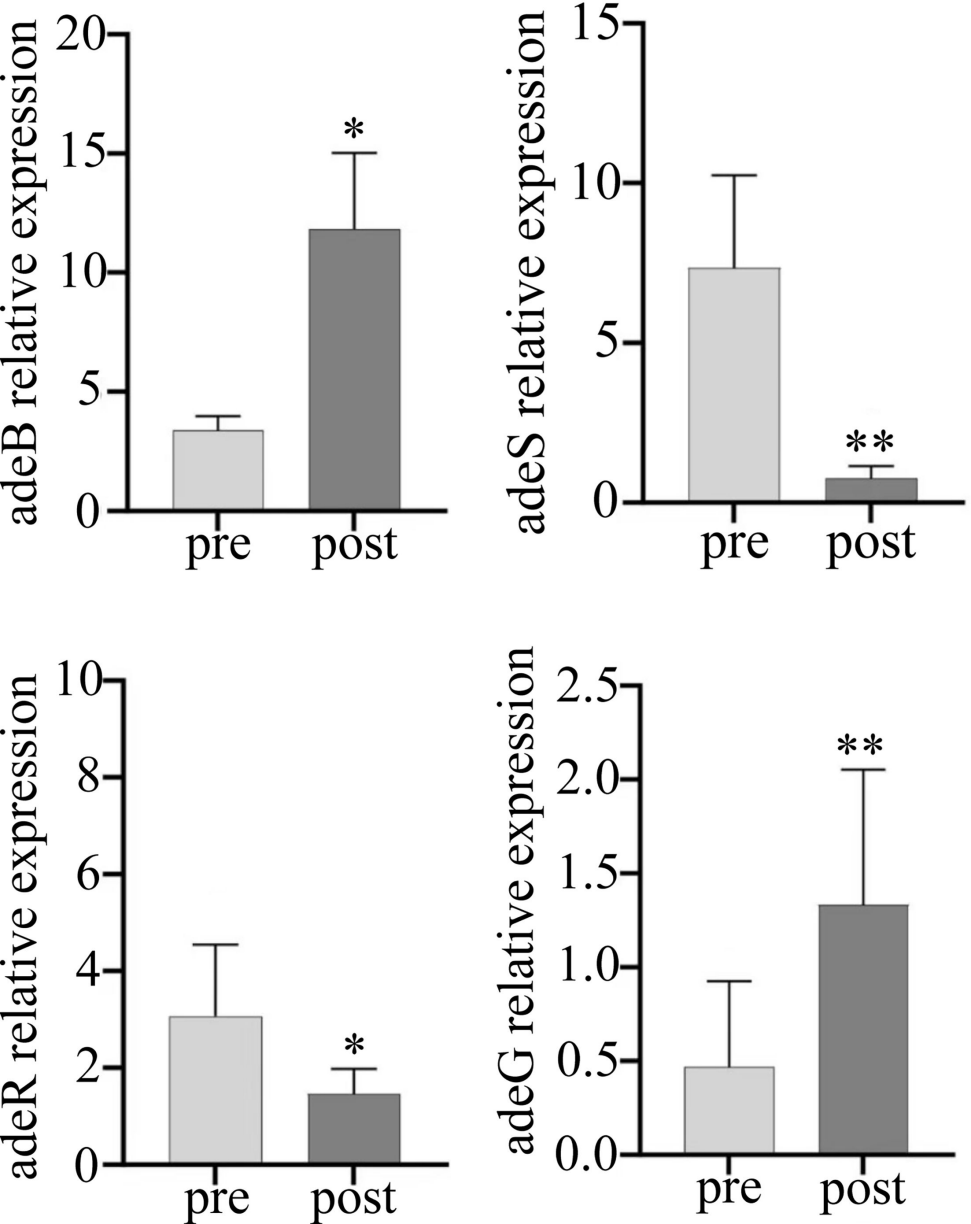
Significant differences in the expression levels of adeS, adeG, adeB, and adeR in 15 strains were observed before (pre-group) and after (post-group) BBH exposure using RT-qPCR. Pre-group vs post-group, *P < 0.05, **P < 0.001. adeB, AdeB efflux pump protein; adeG, AdeG efflux pump protein; adeR, AdeR regulatory protein; adeS, AdeS sensory kinase protein.

After selecting the differentially expressed strains, berberine exposure subcultures were performed, and the relative mRNA expression levels were measured before and after exposure. The results showed that after berberine exposure, the expression of *adeR*, *adeS*, *adeJ*, and *adeM* changed from high expression (RE > 1) to low expression (RE < 1) (Table 5). Statistical analysis indicated that BBH affected the expression levels of efflux pump genes (*adeR*, *adeS*, *adeJ*, *adeM*, *adeG*, and *adeB*) in *A. baumannii*, primarily in four genotypes: *adeS*, *adeG*, *adeB*, and *adeR*. Significant differences in the expression levels of *adeS*, *adeG*, *adeB*, and *adeR* were observed before and after berberine exposure (*P* < 0.05) (Table 6 and Fig. 2).

**TABLE 5:** Median relative expression values of bacterial efflux pump genes before and after berberine exposure^*a*^

	*adeB*	*adeR*	*adeS*	*adeJ*	*adeM*	*adeG*
Before exposure	2.704	1.414	2.808	1.608	11.197	0.003
After exposure	8.282	0.646	0.264	1.376	0.818	0.490

^*a*^ adeB, AdeB efflux pump protein; adeG, AdeG efflux pump protein; adeR, AdeR regulatory protein; adeS, AdeS sensory kinase protein.

**TABLE 6:** Gene expression differences of the efflux pump before and after exposure^*a*^

	*adeB*	*adeR*	*adeS*	*adeJ*	*adeM*	*adeG*
*P* value	0.036	0.036	0.006	0.427	0.078	0.011

^*a*^ adeB, AdeB efflux pump protein; adeG, AdeG efflux pump protein; adeR, AdeR regulatory protein; adeS, AdeS sensory kinase protein.

### Transcriptome sequencing

**Fig 3:**
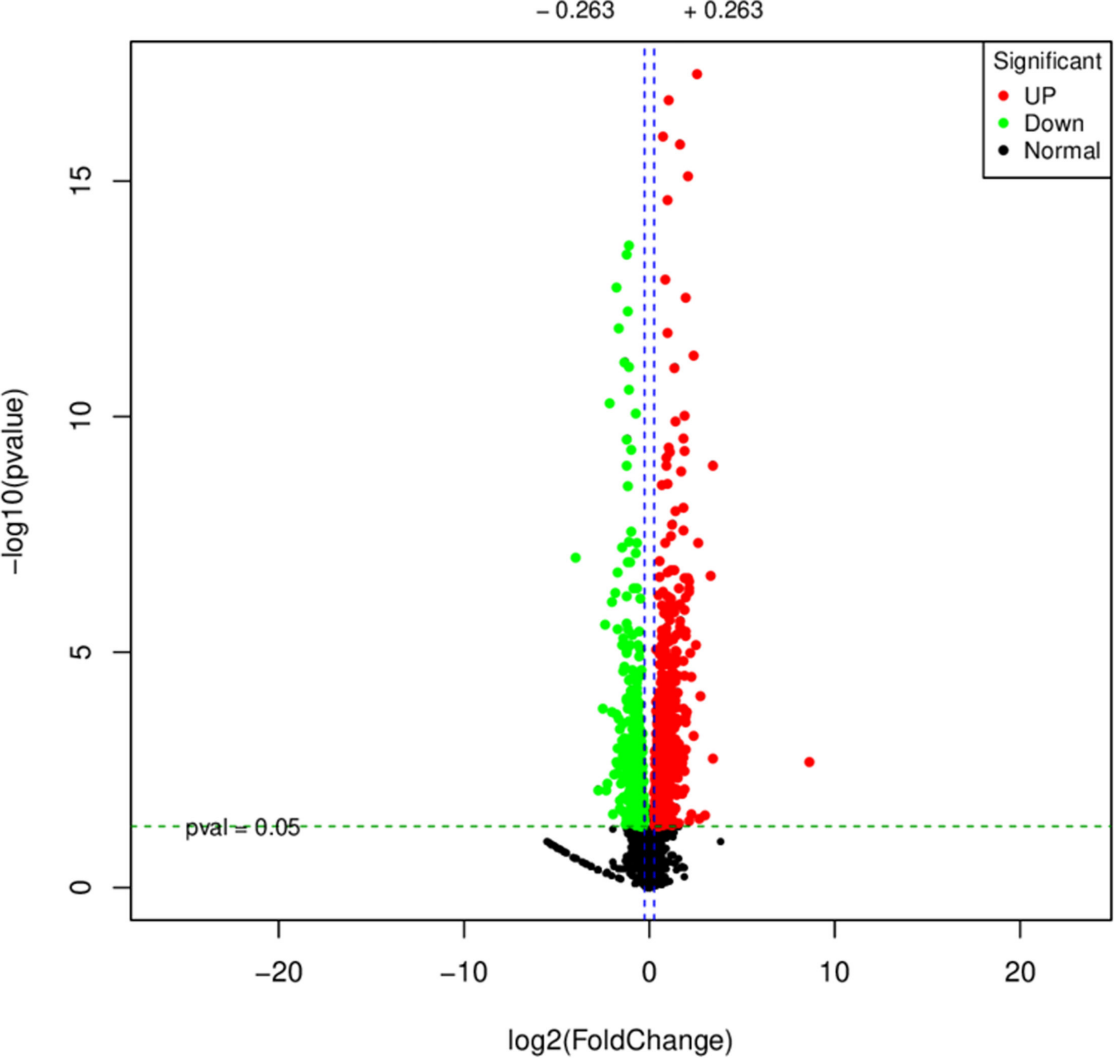
Volcano plot of differential genes (pre-induction vs post-induction). Genes with significantly differential expression are represented by red dots (up-regulated) and green dots (down-regulated), while genes without significantly differential expression are represented by gray dots.

**Fig 4:**
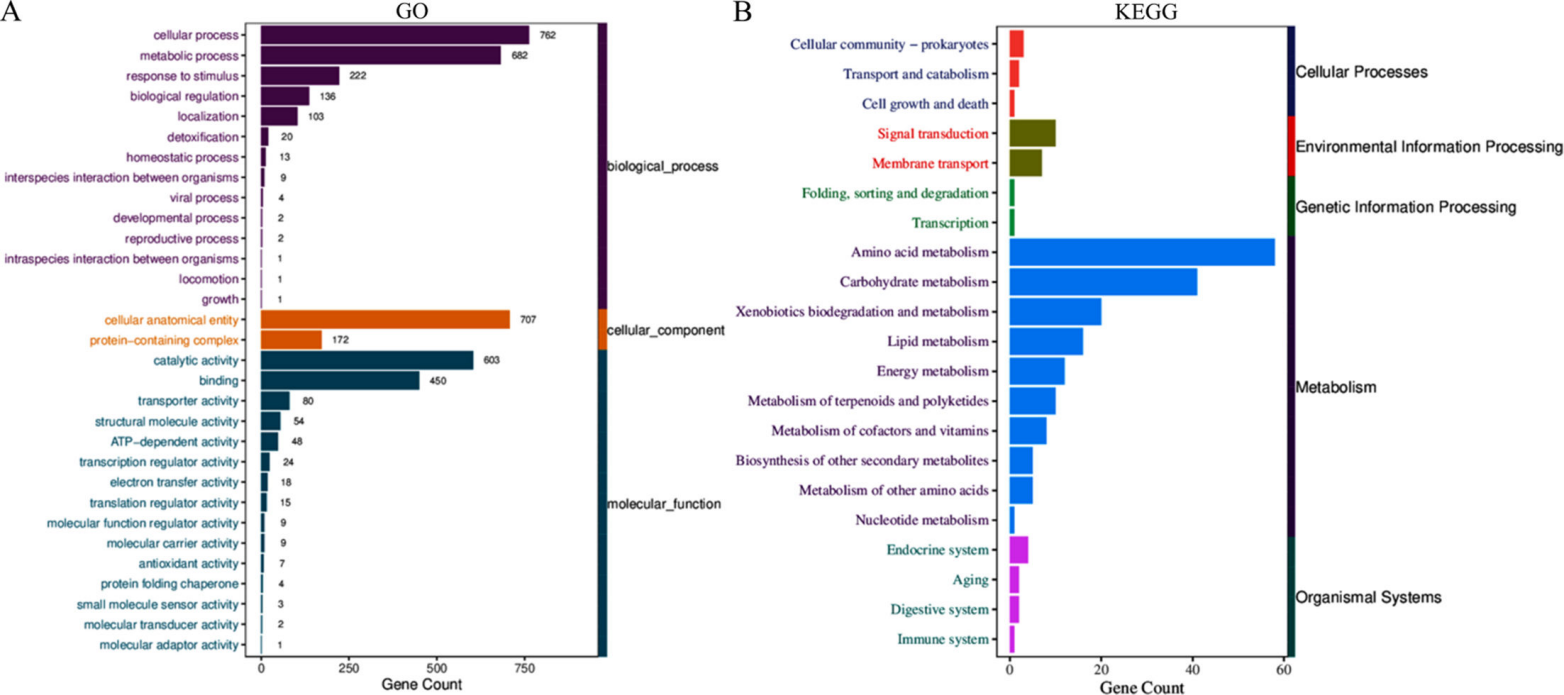
Gene Ontology (GO) and Kyoto Encyclopedia of Genes and Genomes (KEGG) pathway analyses were performed on the differentially expressed genes to elucidate their functional enrichment and biological pathways. (**A**) Gene Ontology analysis identified significantly enriched Gene Ontology terms, with biological processes accounting for 47%, molecular functions accounting for 32%, and cellular components accounting for 21%. (**B**) KEGG analysis identified xenobiotics biodegradation and metabolism as the core pathway for antimicrobial resistance. Other pathways, including membrane transport, folding/sorting/degradation, and cellular community processes, contribute to resistance through mechanisms, such as efflux pumps, target modification, and quorum sensing.

During the detection of differentially expressed genes, with fold change ≥2 and *P*_adj_ <0.05 set as the screening criteria, the results showed that 991 genes were differentially expressed in MDR strains before and after exposure, among which 538 genes had upregulated expression and 453 genes had downregulated expression (Fig. 3). Transcriptome analysis revealed 14 efflux pump-related genes: *adeB*, *adeA*, *adeR*, *adeS*, *adeT1*, *adeN*, *adeL*, *adeF*, *adeG*, *adeH*, *adeI*, *adeJ*, *adeK*, and *adeT2*. Among these, *adeA*, *adeN*, and *adeT2* were the most significantly expressed. Gene Ontology analysis identified significantly enriched Gene Ontology terms, with biological processes accounting for 47%, molecular functions accounting for 32%, and cellular components accounting for 21% (Fig. 4). Among the biological processes, key pathways related to efflux regulation and stress adaptation were notably enriched, including response to stimulus, detoxification, and biological regulation (9). In particular, the activity of transport proteins and ATP-dependent active efflux of antibacterial compounds was a common mechanism of multidrug resistance (10). Additionally, metabolic process and catalytic activity were significantly upregulated, suggesting metabolic adaptation that may facilitate energy provision for efflux pump operation and membrane stabilization (11). Kyoto Encyclopedia of Genes and Genomes (KEGG) analysis results showed that xenobiotics biodegradation and metabolism was the core pathway directly associated with antimicrobial resistance (12). Membrane transport participated in the formation and regulation of antimicrobial resistance through the drug efflux pump mechanism, folding, sorting, and degradation through drug target modification or degradation processes, and cellular community—prokaryotes through quorum sensing-mediated horizontal transfer of drug resistance genes jointly participated in the formation and regulation of antimicrobial resistance (13).

## DISCUSSION

*Acinetobacter baumannii* is a bacterium that acquires resistance to multiple antibiotics. Recent developments in MDRAB strains have made them important human pathogens (14). Carbapenems serve as the first-line therapy for *A. baumannii* infections. However, the global increase in carbapenem resistance poses substantial healthcare costs, economic burden, and public health risks (15). *Acinetobacter baumannii* pathogenesis relies on multiple virulence factors, including porins, lipopolysaccharides, enzymes, biofilm formation, motility, and iron acquisition systems, which enable bacterial survival under stressful conditions and promote severe infections (16). BBH, an emerging therapeutic agent, has recently been explored for the treatment of *A. baumannii* infections. We evaluated the combined efficacy of BBH and clinically relevant antibiotics against MDRAB and investigated BBH-mediated inhibition of efflux pumps to identify novel drugs against *A. baumannii* infections.

Commonly used antibacterial agents, such as penicillins, cephalosporins, tetracyclines, aminoglycosides, and quinolones, are largely ineffective in treating MDRAB. Therefore, there is an urgent need to identify novel antibacterial agents against MDRAB. Over the past two decades, only five new antibiotic classes have been approved, none of which target gram-negative pathogens (4). With the ongoing development and in-depth research on TCM, many Chinese herbal medicines with antibacterial properties have been developed. Due to the highly complex active components of TCM, these medicines not only exert antibacterial effects but also enhance their efficacy by modulating the body’s overall functions (17). Berberine is an alkaloid extracted from Sankezhen, Phellodendri Chinensis Cortex, and Coptidis rhizoma. BBH, a commonly used berberine, exhibits remarkable antibacterial activity. This study showed that BBH synergized with nine antibiotics (amikacin, tetracycline, ceftazidime, ciprofloxacin, cefepime, imipenem, meropenem, levofloxacin, and tigecycline). Notable reductions in MIC were observed, particularly for levofloxacin, with some multidrug-resistant strains reverting to sensitivity. Similar findings were reported by Xiao et al. (18), who observed a decrease in the MIC of ciprofloxacin (32 to 1 mg/L), sulbactam (64 to 4 mg/L), and meropenem (128 to 2 mg/L) when combined with BBH. This phenomenon indicates that BBH may reverse antibiotic resistance or enhance the sensitivity of *A. baumannii* to ineffective antibiotics. Therefore, BBH may serve as an effective adjuvant for the clinical treatment of patients with MDRAB infections, thereby addressing this therapeutic dilemma.

Efflux pumps play a critical role in the development of acquired multidrug resistance. Evaluating the presence of efflux pump genes is essential to curb the spread of antibiotic resistance and formulate appropriate treatment regimens for infected patients. The high prevalence of genes encoding efflux pumps in this bacterium is a key factor in the dissemination of antibiotic resistance among isolates from different geographical regions (19). Overexpression of the AdeABC efflux pump is associated with resistance to carbapenems and cephalosporins in *A. baumannii*. As a tripartite efflux pump belonging to the resistance-nodulation-division family, AdeABC comprises the AdeB component (responsible for extruding antibiotics from the cell), AdeA membrane fusion protein, and AdeC outer membrane protein, the expression of which is regulated by the AdeRS two-component system (20). Point mutations in the AdeRS operon modulate AdeABC activity, leading to resistance to multiple antibiotics, including aminoglycosides, β-lactams, and fluoroquinolones. The resistance-nodulation-division family also includes AdeFGH and AdeIJK efflux pumps, which are associated with tigecycline resistance and are regulated by the transcriptional factors AdeL and AdeN (21). Confronted with severe bacterial resistance, researchers have emphasized the urgent need to develop novel antimicrobial strategies rather than relying solely on traditional antibiotics or new drug development (10). Herbal therapies have emerged as alternatives to antibiotics. Herbal medicines and their secondary metabolites, such as BBH, curcumin, and curcusinol, exhibit potential activity against various MDR bacteria. Additionally, antimicrobial compounds derived from medicinal plants offer advantages, including reduced adverse reactions, improved patient tolerance, low cost, wide acceptance owing to long-term use, renewability, and generally high stability and bioavailability (22). Lin et al. (23) demonstrated that 128 μg/mL BBH inhibits polysaccharide intercellular adhesin synthesis, and 16 μg/mL BBH effectively suppresses extracellular DNA release, thereby inhibiting *Staphylococcus aureus* biofilm formation. Recently, Foong et al. (24) observed that the *tet(A*) gene enhances tigecycline efflux in *A. baumannii* through a synergistic mechanism involving AdeABC and AdeIJK pumps. In this study, the relative mRNA expression levels of efflux pump genes were determined in the sensitive and resistant groups. Significant differences in *adeJ* and *adeB* expression were observed between the groups, suggesting that resistance in local *A. baumannii* isolates may be primarily regulated by these two efflux pump genes. Their overexpression enhances antibiotic efflux and contributes to multidrug resistance in *A. baumannii*. However, the roles of other factors and mechanisms in the development of resistance should not be overlooked. Based on the combined drug susceptibility test results, BBH was confirmed to enhance antibiotic sensitivity *in vitro*. To investigate whether the reduction in MDRAB resistance involved efflux pump regulation, the expression levels of efflux pump genes in MDRAB were compared before and after BBH exposure. Reverse transcription PCR results showed significant differences in *adeS*, *adeG*, *adeB*, and *adeR* expression, with *adeB* exhibiting prominent differences across the sensitive, resistant, exposure, and non-exposure groups. These findings are consistent with those reported by Li et al. (18). Our study confirmed that BBH significantly upregulated *adeB* expression and had a higher affinity for AdeB than for antibiotics, indicating that BBH enhances antibiotic efficacy by competing for AdeB-binding sites. Transcriptome analysis identified 14 efflux pump-related genes, with *adeA*, *adeN*, and *adeT2* showing significant expression levels. Discrepancies between the transcriptome and reverse transcription PCR results may stem from variations in strain passage or small sample sizes, necessitating the use of large cohorts to improve reproducibility and accuracy. Nevertheless, transcriptome data still suggest efflux pump gene regulation in *A. baumannii* resistance and the impact of BBH on gene expression. Notably, we did not cover all efflux pump genes, and BBH may serve as a substrate for other pumps, potentially explaining its synergistic effect with antibiotics and its antibiotic-sensitizing activity in *A. baumannii*.

BBH shows unique synergistic effects compared to natural adjuvants like curcumin and epigallocatechin gallate, which often act via anti-inflammatory or antioxidant properties. For example, Augostine, Catheryn R et al. (25) reported that a high-throughput screening approach with yeast revealed 34 potential synergies from 800 combinations of a diverse natural products (NP) library with four selected NPs of interest (eugenol, β-escin, curcumin, and berberine hydrochloride). The FIC index of both the eugenol + BBH and pterostilbene + BBH combination was ≤0.5, suggesting that these two combinations could synergistically inhibit a series of fungi. In addition, BBH emerged as a promising potential efflux pump inhibitor against multidrug-resistant pathogens. Some studies demonstrate its ability to target diverse efflux pump systems through mechanisms, such as direct binding to pump proteins, downregulation of efflux-related gene expression, and competitive inhibition of substrate binding sites, that enable BBR to synergistically enhance the efficacy of conventional antibiotics by increasing their intracellular accumulation, reducing MICs, reversing antibiotic resistance in MDR clinical isolates, and inhibiting biofilm formation (26–28).

We preliminarily confirmed that BBH enhances the *in vitro* antibiotic susceptibility of MDRAB and demonstrated its modulation of *A. baumannii* resistance via the regulation of efflux pump genes. Although the *in vitro* results are clear and significant, additional *in vivo* studies are warranted to confirm the clinical efficacy of combination therapy. In addition, studies examining the role of BBH in MDRAB infections are limited, and our findings are limited to *in vitro* experiments. Moreover, our findings might be influenced by a single institution, so it is recommended that future multi-center studies and genomic typing based on methods, such as MLST, be conducted to verify the universality and reproducibility of our results. What’s more, further in-depth investigations into the therapeutic efficacy, mechanisms, and animal models are also essential to expand the frontiers of TCM-based bacterial therapies.

### Conclusion

The remarkable adaptability of *A. baumannii*, coupled with its multiple mechanisms for acquiring and transferring antibiotic resistance determinants, has posed enormous challenges to current treatment, hastened the arrival of the “post-antibiotic era,” and underscored the urgency of developing novel therapeutic approaches (29). TCM has emerged as a promising avenue, as traditional antibiotics have proven inadequate for addressing bacterial resistance. We evaluated the antibacterial activity of BBH in combination with several commonly used antibiotics in clinics and demonstrated that BBH can ameliorate drug resistance in *A. baumannii* by regulating efflux pump gene expression. These findings provide a theoretical foundation for new strategies and methodologies to improve the cure rate of MDRAB infections and may inform future *in vivo* studies for the treatment of infectious diseases.

## Data Availability

The raw data are available in NCBI under BioProject number PRJNA1280536, and the sample accession numbers are SRX29281331 to SRX29281338.

## SUPPLEMENTAL MATERIAL

Table S1 spectrum.02565-25-s0001.xlsx Specific MIC and FIC values for each strain and each drug.

ASM does not own the copyrights to Supplemental Material that may be linked to, or accessed through, an article. The authors have granted ASM a non-exclusive, world-wide license to publish the Supplemental Material files. Please contact the corresponding author directly for reuse.
